# Selective Homogeneous Assay for Circulating Endopeptidase Fibroblast Activation Protein (FAP)

**DOI:** 10.1038/s41598-017-12900-8

**Published:** 2017-10-02

**Authors:** Travis W. Bainbridge, Diana Ronai Dunshee, Noelyn M. Kljavin, Nicholas J. Skelton, Junichiro Sonoda, James A. Ernst

**Affiliations:** 1 Protein Chemistry, Genentech Inc., South San Francisco, CA 94080 USA; 2 Molecular Biology, Genentech Inc., South San Francisco, CA 94080 USA; 3 Molecular Oncology, Genentech Inc., South San Francisco, CA 94080 USA; 4 Discovery Chemistry, Genentech Inc., South San Francisco, CA 94080 USA; 5 Cancer Immunology, Genentech Inc., South San Francisco, CA 94080 USA; 6 Neuroscience, Genentech Inc., South San Francisco, CA 94080 USA

## Abstract

Fibroblast Activation Protein (FAP) is a membrane-bound serine protease whose expression is often elevated in activated fibroblasts associated with tissue remodeling in various common diseases such as cancer, arthritis and fibrosis. Like the closely related dipeptidyl peptidase DPPIV, the extracellular domain of FAP can be released into circulation as a functional enzyme, and limited studies suggest that the circulating level of FAP correlates with the degree of tissue fibrosis. Here we describe a novel homogeneous fluorescence intensity assay for circulating FAP activity based on a recently identified natural substrate, FGF21. This assay is unique in that it can effectively distinguish endopeptidase activity of FAP from that of other related enzymes such as prolyl endopeptidase (PREP) and was validated using *Fap*-deficient mice. Structural modeling was used to elucidate the mechanistic basis for the observed specificity in substrate recognition by FAP, but not by DPPIV or PREP. Finally, the assay was used to detect elevated FAP activity in human patients diagnosed with liver cirrhosis and to determine the effectiveness of a chemical inhibitor for FAP in mice. We propose that the assay presented here could thus be utilized for diagnosis of FAP-related pathologies and for the therapeutic development of FAP inhibitors.

## Introduction

Fibroblast activation protein, (FAP, also known as FAPα, seprase, or circulating antiplasmin-cleaving enzyme, (EC 3.4.21.B28) is a type II transmembrane serine protease and a member of the S9 family of proline-specific proteases which includes dipeptidyl peptidase IV (DPPIV), DPP8, DPP9, prolyl endopeptidase (PREP, also known as POP) (1–4). FAP was originally identified as a protein highly expressed on the surface of the activated stromal fibroblasts in tumors^1–3^. In adult mammals, elevated FAP expression in activated fibroblasts is associated with remodeling of tissues at sites of inflammation in disease, including several forms of cancer^4–8^, rheumatoid arthritis and osteoarthritis^9,10^, liver disease^11–14^, inflammatory bowel diseases^15,16^ and idiopathic pulmonary fibrosis^17,18^. Furthermore, stromal expression of FAP is linked to immunosuppressive tumor microenvironment and poor prognosis in various cancers, suggesting a role in cancer progression and anti-tumor immunity^4–8,19^. Although the contribution of FAP in the development of these diseases is not well understood, some preclinical studies support the role of FAP in determining disease severity^8,20,21^.

Despite intense studies over the past two decades, the identities of physiological substrates for FAP remain elusive. FAP, like its closest relative, DPPIV, exhibits dipeptidyl peptidase activity, cleaving after the proline present at the second position from the N-terminus in various secreted proteins and peptide hormones^22^. FAP, but not DPPIV, also possesses endopeptidase activity, preferentially cleaving carboxy-terminal to a Gly-Pro sequence^23,24^. The Gly-Pro requirement results from the unique property of glycine to adopt a positive phi dihedral angle, allowing the P_3_ residue to avoid steric clashes with the protease^23,25^. While collagens have repeating Gly-Pro motifs, they are resistant to degradation by FAP except in a denatured or partially processed form (e.g., gelatin)^26^. Consistent with the role of FAP in regulating collagen turnover, *Fap* KO mice show elevated collagen levels in pathogenic conditions^20,21^. FAP has also been proposed to cleave at a specific site near the N-terminus of α2-Antiplasmin (α2AP) to potentiate its activity^27^. Fibroblast growth factor 21 (FGF21) is a metabolic hormone that signals through the βKlotho and FGFR coreceptors and activation of this pathway is under investigation for the treatment of metabolic diseases, such as type 2 diabetes and non-alcoholic steatohepatitis^28^. We and others have recently found that FAP also cleaves FGF21 at a specific site proximal to the C-terminus, leading to its inactivation, as this region of the molecule is crucial for binding βKlotho^29–31^. In both α2AP and FGF21, the specific cleavage site targeted by FAP possesses the consensus Gly-Pro sequence at P2-P1 position, and these amino acid residues are essential for cleavage by FAP^32^.

Although FAP is produced as a membrane-bound protein, the extracellular domain encoding the active enzyme can be shed from the cell surface, and therefore soluble FAP protein is readily detectable in serum and plasma by a standard sandwich ELISA. The level of FAP protein has been shown to be elevated in patients with cirrhosis^12,33,34^, suggesting a diagnostic value to measuring circulating FAP levels in these and perhaps other diseases. Another approach to measure FAP protein levels is based on enzymatic activity, in contrast to the activity-independent ELISA. FAP protein can be isolated from tissue or blood samples by immunocapture with an FAP-specific antibody, followed by a general fluorescence intensity assay for dipeptidyl-peptidases using a peptide substrate attached to a chemically quenched dye, such as Z-Gly-Pro-7-amido-4-methylcoumarin (AMC) or Ala-Pro-7-amino-4-trifluoromethyl-coumarin (AFC)^13,21,35,36^. The immunocapture step is necessary to eliminate other related DPP enzymes from the reaction. Alternatively, endopeptidase substrates (e.g. Acetyl-Ala-Gly-Pro-AFC, MEPLGRQLTSGP-AMC, etc.) containing the consensus Gly-Pro dipeptide have been used without immunocapture^37–39^. These substrates are likely targeted also by other circulating proline-specific endopeptidases such as PREP that could be present in the reaction. Nonetheless, this type of assay could be appropriate for characterization of a broad S9 protease inhibitor such as Talabostat (also known as Val-boro-Pro or PT100)^37,38^. A fluorescence-based homogeneous assay reagent to specifically monitor FAP activity called 3144-AMC, ARI-3144 or “N-terminally blocked FAP specific substrate” has also been described by William Bachovchin and his collaborators^11,14,29,40^. However, the identity of this substrate remains unpublished and not readily available to a broader community. Here we describe a novel homogeneous fluorescence intensity assay for circulating FAP activity. This assay utilizes a modified peptide substrate based on the endopeptidase cleavage site of FGF21, a newly identified natural substrate for FAP, in a quenched dye format and is selective for FAP.

## Results

### An FGF21-based quenched-fluorescence peptide is cleaved by FAP and PREP

**Figure 1:**
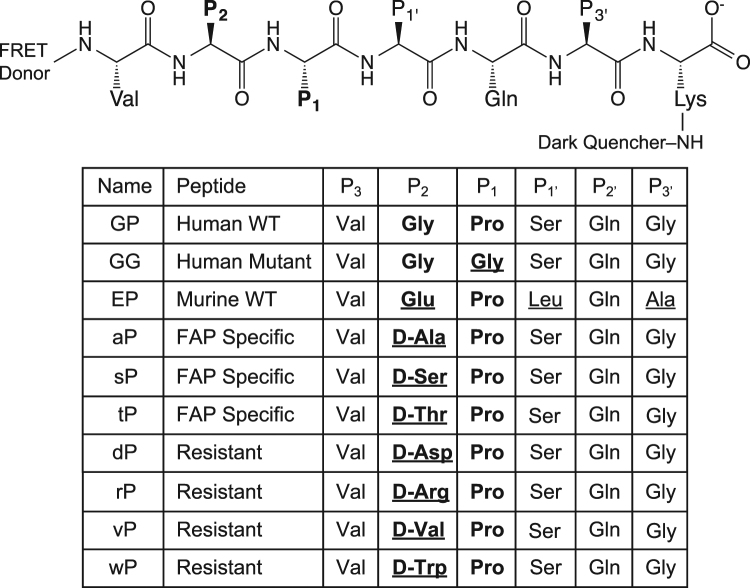
Design of fluorescence-quenched peptides. Peptides contain an N-terminal fluorescent donor, followed by six amino acid residues of the region flanking the dominant FAP endopeptidase cleavage site of human FGF21, ending with an additional C-terminal lysine conjugated to a dark quencher. Variants include substitution of the P_1_ proline with glycine, the P_2_ glycine with D-alanine or the entire homologous region of murine FGF21.

**Figure 2:**
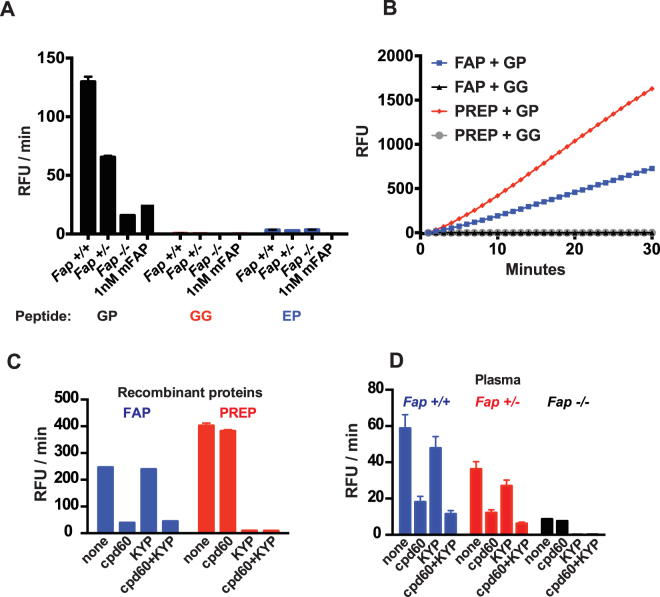
Plasma cleavage of fluorescence-quenched peptides. (**A**) Cleavage rate of human WT (GP), human mutant (GG) and murine (EP) FGF21-based peptides in plasma samples isolated from *Fap* ^+/+^, *Fap* ^+/−^, and *Fap* ^−/−^ mice, as indicated (N = 3 mice per genotype). As a control, the cleavage reaction was run with 1 nM recombinant mouse FAP. Concentration of peptide substrates in this experiment was 3 µM. (**B**) Cleavage rate of the GP peptide by recombinant human FAP or PREP proteins at 1 nM enzyme concentration. (**C**) Cleavage rate of the GP peptide by recombinant human FAP (left, blue) or PREP (right, red) in the presence of an FAP-specific inhibitor cpd60 and/or PREP-specific inhibitor KYP-2047 (KYP). (**D**) Cleavage rate of the GP peptide in plasma from *Fap* ^+/+^ (blue), *Fap* ^+/−^ (red), and *Fap* ^−/−^ (black) mice in the presence of cpd60 and/or KYP (N = 3 per group).

A peptide containing the six amino acid residues surrounding the FAP cleavage site near the C-terminus of human FGF21, termed the “GP” probe, (Fig. 1) was synthesized for the purpose of monitoring FAP endopeptidase activity. The peptide is flanked by a FRET-donor (HyLite Fluor 488) and a dark quencher (QXL 520). By design, fluorescence intensity is suppressed due to the close proximity of the quencher dye to the donor fluorophore, and it is liberated by protease-catalyzed cleavage of the peptide. As controls, variant peptides containing a substitution of the P_1_ proline with glycine (“GG” probe) or the homologous region of murine FGF21 (“EP” probe) were also generated. Both control probes lack the Gly-Pro consensus necessary for FAP-based cleavage, thus serve as negative controls (Fig. 1). These three peptides were used to evaluate the FAP endopeptidase activity in the plasma of wild type (WT), heterozygous and *Fap*-deficient (KO) mice. We have previously demonstrated by immunoblot analysis that FAP is present in the plasma from WT mice, to a lesser degree in heterozygotes and undetectable in KO mice^29^. When the GP probe was used, plasma from WT mice produced a strong signal, indicating efficient cleavage of the probe (Fig. 2A). As expected, plasma from *Fap* heterozygous mice exhibited approximately half the activity as that from WT mice, and that from homozygous *Fap* KO mice exhibited even lower, but significant activity. When plasma from WT mice was tested using GG and EP probes, no and a minimal signal was obtained respectively. Furthermore, purified recombinant mouse FAP protein cleaved the GP but not GG or EP probes (Fig. 2A).

The ability of *Fap* KO mouse plasma to cleave the GP probe and to a much lesser degree, the EP probe, but not the GG probe, suggested the presence of other proline-specific endopeptidases in plasma. Although prolyl endopeptidase (PREP) is predominantly a cytosolic enzyme, it is known to be present at variable levels in plasma^41,42^ and seemed a likely candidate for the source of residual probe cleavage by the KO plasma. Indeed, when 1 nM of recombinant human FAP or PREP was tested, both enzymes readily cleaved the GP peptide, while leaving the mutant GG peptide intact (Fig. 2B). To further investigate the involvement of PREP in the cleavage of the GP probe, we used an FAP-selective inhibitor, cpd60^30,43^, and a PREP-selective inhibitor KYP-2047^44,45^ (Supplementary Fig. 1). Both inhibitors were tested at a concentration that provides selective inhibition of each enzyme (Fig. 2C). Using these inhibitors, we found that the residual GP probe cleaving activity in *Fap* KO sample was sensitive to KYP, but not cpd60 (Fig. 2D). At the same time, the GP probe activity in WT or heterozygous samples were clearly sensitive to cpd60. Taken together, we concluded that FAP is the major enzyme responsible for the cleavage of the GP probe in plasma, while PREP has a smaller but significant contribution.

### Impact of P_2_ D-Ala substitution on FAP cleavage kinetics

**Figure 3:**
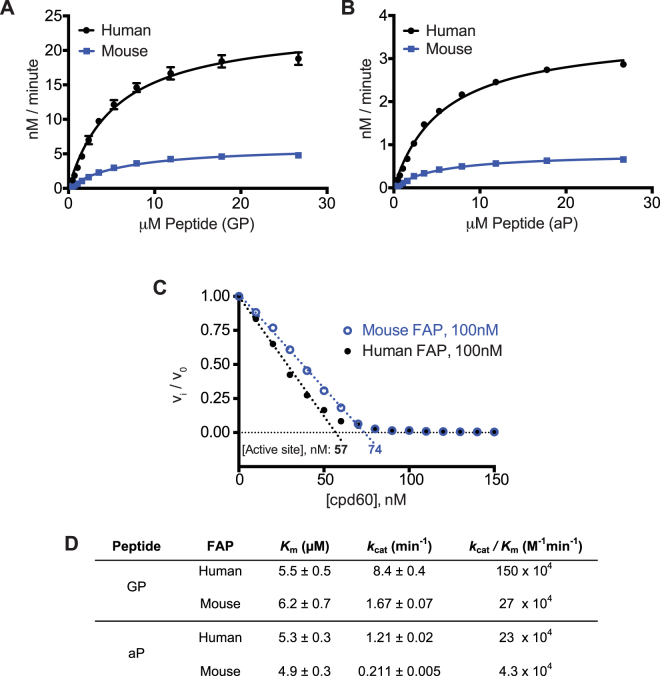
Cleavage kinetics of GP and aP substrates by recombinant FAP. (**A** and **B**) Michaelis-Menten saturation curves for human (black) or mouse (blue) recombinant FAP cleavage of (**A**) the GP or (**B**) aP peptides. (**C**) Plot of fractional velocity as a function of cpd60 concentration, for recombinant mouse or human FAP with cpd60, using GP peptide. Mouse and human FAP preparations were found to be 74% and 57% active, respectively. (**D**) Human and mouse FAP enzyme kinetics constants with the GP and aP substrates, corrected for experimentally determined active enzyme concentrations determined in (**C**). Error is SEM, determined from three experimental replicates. FAP molar concentrations are based on monomer for active site titration and kinetics calculations.

FAP has previously been shown to tolerate a change of the consensus glycine to a D-alanine at position P_2_ in a α2AP-based peptide substrate, although a weaker catalytic efficiency was observed relative to the parental peptide due to a decrease in *k*_*cat*_ of approximately eight-fold^23^. In the context of FAP inhibitors, this substitution has been reported to impart specificity for FAP over PREP^40^. In an attempt to increase the specificity of our assay, we incorporated this substitution into the FGF21-based GP peptide to produce the “aP” probe (Fig. 1). For an activity probe to be of practical use for a diagnostic purpose, it is important that this change does not have a strong negative impact on the enzyme kinetics. We, therefore, compared the kinetics for both the GP and aP peptides, using both human and mouse FAP proteins (Fig. 3A,B & D).

Interestingly, mouse FAP exhibited a lower catalytic efficiency than human FAP, mainly due to a reduced *k*_*cat*_ (Fig. 3A & B). From the kinetics data alone, it was not clear if this is an innate efficiency difference between the orthologs or if it was due to differences in active enzyme concentration between the two preparations. To address this question, an active site titration was performed, using the tight binding inhibitor, cpd60 (Fig. 3C). We found that the preparation of mouse FAP had, in fact, a higher relative concentration of active enzyme, confirming its intrinsically weaker catalytic efficiency. The kinetics data shown in Fig. 3D are thus corrected for the experimentally determined active enzyme concentration. The *K*_*m*_ value is essentially unchanged between the parental (GP) and aP peptides, and we observed the expected seven to eight-fold decrease in *k*_*cat*_ for aP cleavage^25^.

### P_2_ D-Ala substitution provides FAP specificity

**Figure 4:**
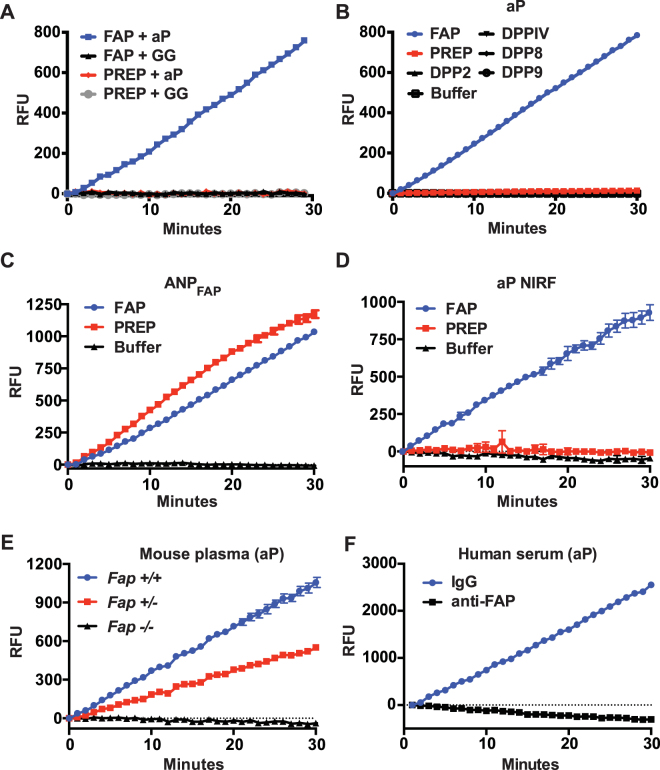
Specificity of aP peptide cleavage. (**A**) Cleavage rate of the aP or GG peptide by 1 nM recombinant, human FAP or PREP. (**B**) Cleavage rate of the aP peptide by a panel of recombinant, human prolyl peptidases. Each recombinant peptidase was used at 2.5 µg/ml. (**C** and **D**) Cleavage rate of ANP_FAP_ (**C**) or aP NIRF (**D**) by 10 nM recombinant, human FAP or PREP. (**E**) Cleavage rate of the aP peptide in plasma from *Fap* ^+/+^, *Fap* ^+/−^, and *Fap* ^−/−^ mice. Plasma was diluted 10-fold. (**F**) Cleavage rate of the aP peptide in anti-FAP or control IgG-immunodepleted human serum. Serum was diluted 2.5-fold. Complete FAP removal following anti-FAP immunodepletion was shown previously by immunoblotting^29^.

Encouraged by the kinetics data, we decided to test the specificity of the aP probe against PREP and a panel of related peptidases. Using a concentration of 1 nM enzyme, cleavage of the aP probe by human FAP is clearly detectable, but not the GG probe (Fig. 4A). In contrast, human PREP is unable to digest either probe. In addition, a broader set of human S9 and S28 peptidases, did not exhibit an ability to cleave the aP probe (Fig. 4B). This is consistent with the general lack of endopeptidase activity of these prolyl dipeptidyl peptidases.

Another internally-quenched FRET peptide substrate (ANP_FAP_) for FAP has recently been reported and demonstrated for use as an activity-based, *in vivo* imaging tool^39^. The peptide sequence contains two internal Gly-Pro dipeptide motifs, susceptible to FAP cleavage and a Cy5.5/QSY21, quenched-FRET pair. To evaluate specificity, the authors tested ANP_FAP_ for cleavage *in vitro* by FAP, DPPIV and MMP-2, but only detected cleavage in the presence of FAP. Our experience with the GP probe led us to suspect that the Gly-Pro motifs in ANP_FAP_ are also recognized by PREP. Therefore, ANP_FAP_ was synthesized and tested for cleavage by recombinant human FAP and PREP (Fig. 4C). As suspected, both FAP and PREP efficiently cleave ANP_FAP_ *in vitro*. While it is currently unclear how much of a liability PREP or other non-specific prolyl endopeptidase activity might be in the context of *in vivo* FAP imaging, we were interested to see if our aP probe would retain its specificity and FAP cleavability if the FRET pair was replaced with near infrared fluorophores (NIRF) more suitable to *in vivo* imaging. A new peptide was generated (aP NIRF), identical to aP, but the FRET donor and quencher were replaced with Cy5.5 and QSY21, respectively. In contrast to ANP_FAP_, we found that FAP, but not PREP, efficiently cleaves aP NIRF (Fig. 4D).

Finally, having demonstrated specificity using several purified recombinant enzymes, we returned to the analysis of the WT and *Fap* KO mice plasma as in Fig. 2A, but using the aP probe. As expected, the aP probe shows a clear signal with WT plasma, intermediate with heterozygous plasma and satisfyingly, the aP probe exhibits no cleavage with *Fap* KO plasma samples (Fig. 4E). Furthermore, the signal is lost after FAP immunodepletion from human serum, but not in control IgG-immunodepleted serum (Fig. 4F).

### Molecular determinants for the substrate specificity of FAP

**Figure 5:**
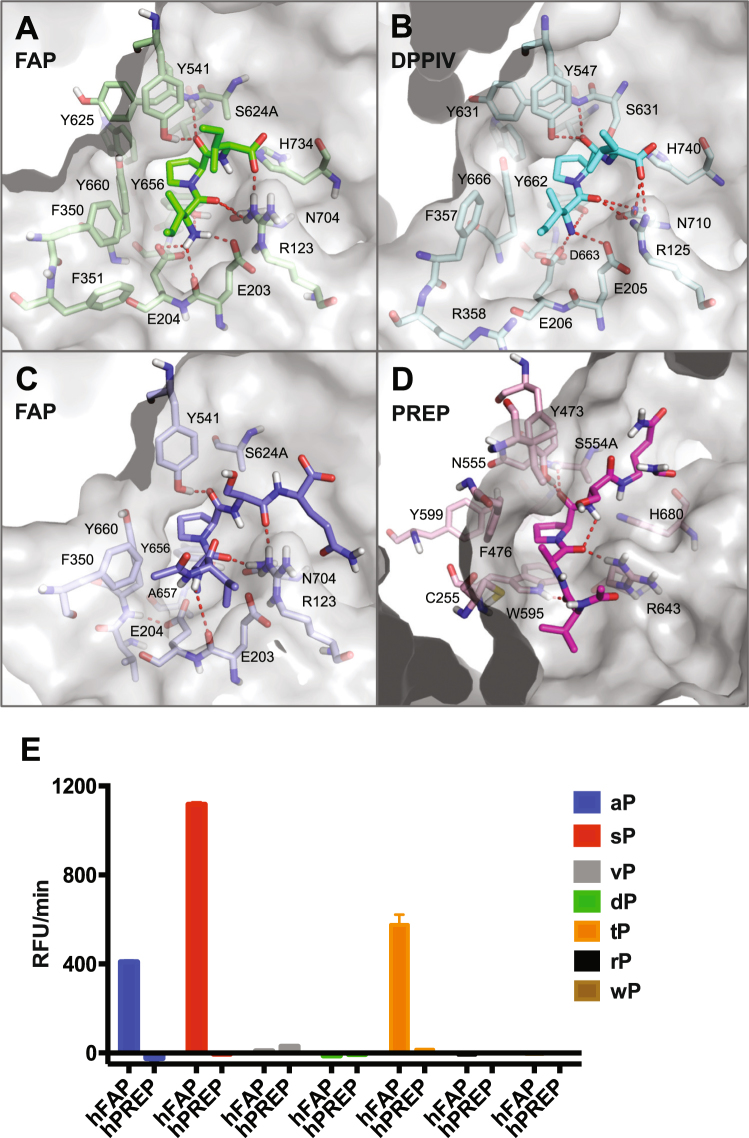
Binding mode of peptide substrates to FAP, DPPIV and PREP. (**A**) FAP active site (grey surface and pale green sticks) bound to modeled IPI substrate tripeptide (green sticks). (**B**) DPPIV active site (grey surface and pale cyan sticks) bound to IPI substrate tripeptide (cyan sticks). (**C**) FAP active site (grey surface and pale blue sticks) bound to modeled acetyl-VaPSQ substrate peptide (blue sticks). (**D**) Representative induced-fit docking model of PREP (grey surface and pale pink sticks) bound to acetyl-VAPSQ-amide substrate peptide (pink sticks). In all panels, red dashed lines indicate intermolecular and intra-substrate hydrogen bonds. (**E**) Comparison of cleavage rates by human FAP on VxPSQG peptide substrates containing various D-enantiomer residues at the P_2_ position (xP).

In order to elucidate the mechanistic basis for the observed aP probe recognition by FAP but not by DPP4 or PREP, a model of peptide-bound FAP was generated by superposition of the apo FAP coordinates onto diprotin A (Ile-Pro-Ile or IPI) tripeptide-bound DPPIV, with the active site side chain of S624 of FAP replaced by an alanine residue^46,47^. This model of FAP shows the coordination of the N-terminal amine at P_2_ (E203, E204 and Y656), coordination of the P_2_ carbonyl oxygen (R123 and N704), positioning of the P_1_ proline side chain in a hydrophobic pocket (Y625, V650, W653, Y656 and Y660), and coordination of the proline P_1_ carbonyl (Y541 and Y625 back bone NH) so as to position the scissile bond adjacent to the catalytic residue S624 (Fig. 5A). As a result of this binding mode, the side chain of the P_2_ residue is directed into the space proximal to F350 and F351. This is highly reminiscent of the substrate binding mode of DPPIV (Fig. 5B), and explains how both enzymes can act as dipeptidases for proteins terminating with an L-amino acid at P_2_ and a proline at P_1_.

Induced fit docking was used to generate hypotheses for how FAP could also act as an endopeptidase. Residues within 5 Å of the IPI peptide were allowed to move during the docking calculation to accommodate the acetyl-VaPSQ substrate peptide. Three of the top 5 scoring ligand poses are able to accommodate the P_2_ side chain of the substrate by a modest reorientation of the E204 side chain towards A657 (χ_1_ changes from 180° to −60° and χ_2_ from +60° to 180°) with the concomitant formation of a hydrogen bond from the E204 carboxylate to the backbone NH of Y660 (Fig. 5C). These three ligand poses all have the substrate P_2_ residue with a positive backbone phi dihedral angle, the amide proton within hydrogen bonding distance of E203 (backbone NH or side chain carboxylate, depending on pose) and the alanine side chain in the pocket of space generated by relocation of the E204 side chain. Importantly, this binding mode readily accommodates acylation of the P_3_ amine with the additional N-terminal residues directed into the large space between the α/β hydrolase and β-propeller domains of FAP. This binding mode explains the ability of FAP to act as an endopeptidase for substrates with small D-amino acids at the P_2_ position. Moreover, such a binding mode would not be possible in DPP4 since A657 is replaced by the larger D663 and hence E206 (the equivalent of E204 in FAP) has no room to reorient, as has been suggested by others^23,25,47^. Indeed, a D663A substitution in DPPIV increases the endopeptidase activity of DPPIV while the corresponding A657D substitution in FAP decreases the endopeptidase activity of this enzyme^47^.

The substrate binding mode of PREP has been revealed in some co-crystal structures in which the active site serine residue has been mutated to alanine^48,49^. Conserved substrate interactions include a hydrogen bond from the P_3_ carbonyl to W595 indole, a hydrogen bond from the P_2_ carbonyl to R643 and an intra-substrate hydrogen bond from the P_2_ carbonyl to the P’_1_ amide NH (Fig. 5D). As a result of this hydrogen bond network, the substrate P_2_ residue is constrained to a have a negative phi backbone dihedral angle with the P_2_ side chain directed towards F476. A D-amino acid at P_2_ would not be accommodated due to the high energy of forcing such a residue to have a negative phi, and a steric clash since the P_2_ side chain would be orientated directly at the side chain of C255. Thus, the specific substrate-binding mode required by PREP prevents it from recognizing and cleaving VxPSQ or other substrates containing a D-amino acid residue at P_2_ position.

To validate the molecular modeling, relative cleavage rates of human FAP and PREP were experimentally determined using substrates containing a variety of D-residues at the P_2_ position (Fig. 5E). We found that FAP can cleave substrates containing D-Ser or D-Thr, in addition to D-Ala, at the P_2_ position. As predicted from the structural modeling, none of these D-residue containing peptides were cleaved by PREP to an appreciable degree.

### The diagnostic utility of aP probe in analyzing FAP activity in blood

**Figure 6:**
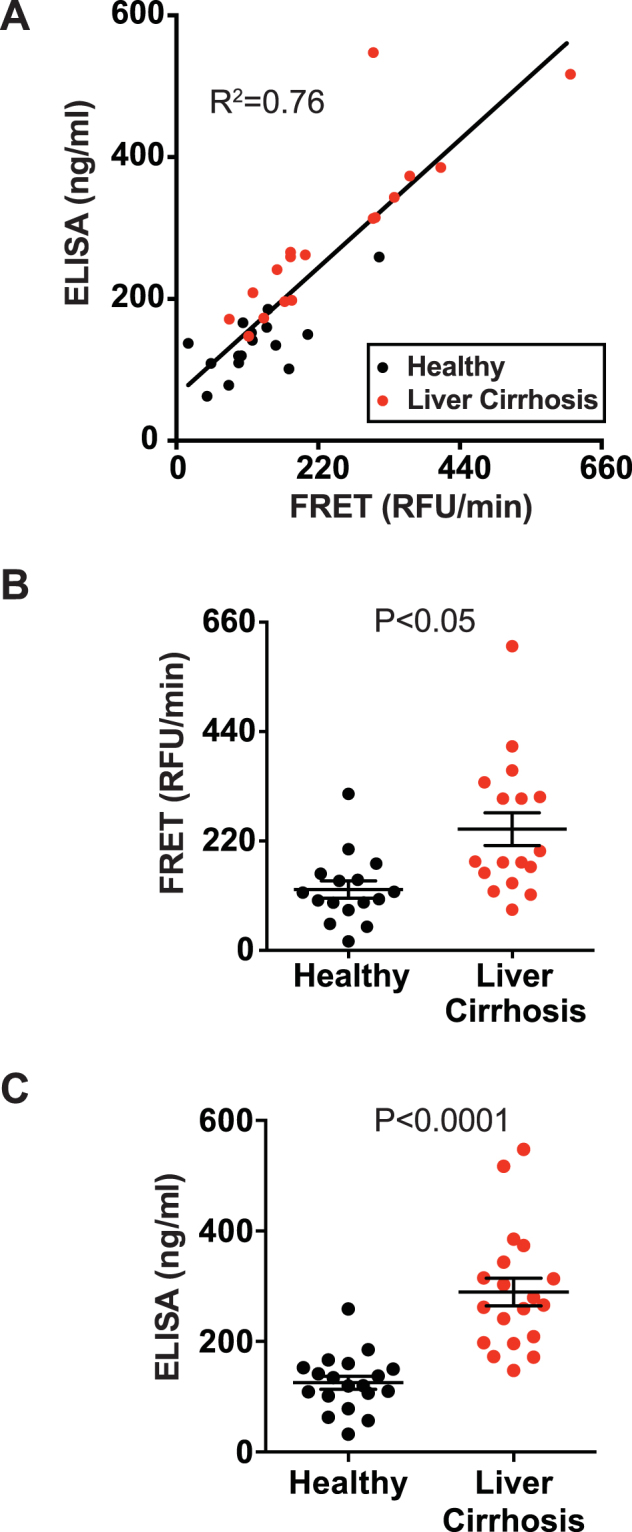
FAP activity in serum correlates with liver disease and FAP levels. (**A**) Scatter plot showing FAP activity levels determined by using the aP peptide (x axis) and FAP protein levels determined by ELISA in serum samples (y axis) from healthy individuals (N = 16, black circles) or individuals previously diagnosed with liver cirrhosis (N = 17, red circles). R^2^ value is indicated in the graph. (**B** and **C**) The same data as (**A**), with FRET and ELISA data presented separately, including mean ± SEM. P values indicated were calculated by student t-test.

Previously, serum FAP levels and activity have been shown to correlate with liver fibrosis^11^. Therefore, we decided to determine if we can recapitulate these findings by the aP probe-based fluorescence assay. We obtained serum samples from healthy volunteers and from individuals who had been diagnosed with non-viral liver cirrhosis. The observed FAP activity correlated well with FAP protein level (R^2^ = 0.76, p values < 0.05) and using the activity assay, we were easily able to detect levels as low as 63 ng/ml (Fig. 6A). When the healthy and disease groups were compared, we found a measurable increase in FAP activity as determined using the aP probe, P < 0.05 (Fig. 6B) and in FAP levels determined by a conventional sandwich ELISA, P < 0.0001 (Fig. 6C).

**Figure 7:**
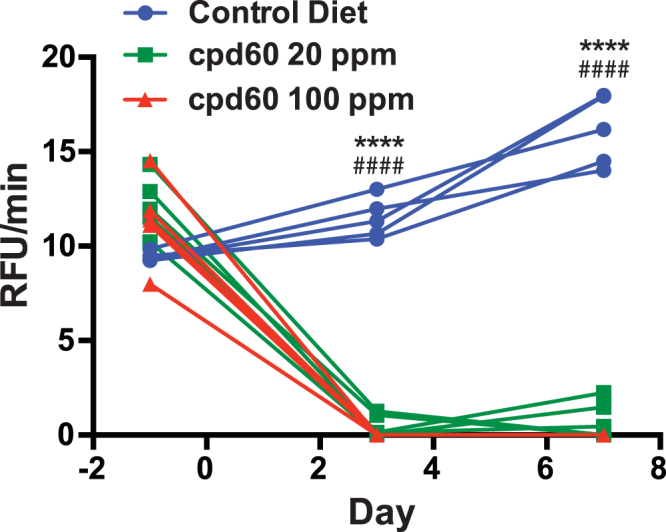
Plasma FAP activity is completely suppressed in mice fed chow with cpd60. Ten to eighteen-week old C57/BL6 background mice were fed *ad libitum* control chow (blue circles), diet containing 20 ppm of cpd60 (green squares), or diet containing 100 ppm of cpd60 (red triangles). Plasma was isolated from blood retrieved at the indicated time points and FAP activity levels determined using the aP peptide. N = 5 animals per group. P values indicated were calculated by student t-test (****P < 0.0001 for 20 ppm cpd60 vs control diet; ^####^P < 0.0001 for 100 ppm cpd60 vs control diet).

We next wanted to determine whether the new aP-based assay can be used to determine the pharmacodynamic activity of FAP inhibitors *in vivo*. To do this, we fed mice with chow containing the FAP inhibitor cpd60 or a control diet without the inhibitor (Fig. 7). The FAP activity was determined in plasma isolated at days 3 and 7 after cpd60 feeding started. The results clearly showed that at 20 ppm, cpd60-feeding could strongly suppress circulating FAP activity in mice, while at 100 ppm suppression was complete. Therefore, we conclude that the aP-based assay can be used to evaluate the pharmacodynamics of FAP inhibitors selectively, without interference from PREP activity potentially present in plasma.

## Discussion

Clinical development of an enzyme inhibitor as a therapeutic agent can be significantly facilitated by the ability to determine the activity of the enzyme in biological samples in a homogenous assay format. First, identification of patients with high levels of active enzyme may support the discovery of a patient sub-population who might benefit most from the use of an inhibitor or an enzyme-targeted anti-tumor agent, such as the recently described anti-DR5/FAP bispecific antibody^50^. This aspect of drug development has become ever more important in the era of precision medicine. In addition, the ability to measure the effect of an inhibitor in clinical trial subjects would enhance the understanding of the relationship between pharmacokinetics and pharmacodynamics; an aspect that is vital in selecting an appropriate dose regimen to balance drug efficacy and safety. In this regard, one major challenge in the clinical development of FAP inhibitors has been the lack of a selective homogenous assay for FAP activity.

The new FRET assay based on an FGF21 peptide containing the D-Ala-Pro dipeptide described here provides several notable advancements. First and foremost, the assay enables the measurement of FAP endopeptidase activity in serum or plasma samples without immunocapture or any other purification steps, even for samples that contain related S9 proteases, most notably PREP. Second, because of its strict specificity, the activity assay for FAP could substitute for the more cumbersome sandwich ELISA, at least for certain applications. Indeed, we observed a good correlation between FAP activity and FAP protein levels in human serum samples from both healthy individuals and patients with cirrhosis (Fig. 6). Thus, the FAP activity assay could save time and cost for investigators who are interested in determining FAP levels in samples that do not contain FAP inhibitors. Finally, the aP probe can be synthesized from commonly used fluorescent dyes and amino acids without any specialized methods. This means that the probe can be ordered from a peptide probe vendor through a custom synthesis request. This increases the accessibility, practicality and utility of the new assay method.

As a practical application of the new assay, we demonstrated that an FAP-selective inhibitor cpd60^38^ can be used *in vivo* to suppress the activity of circulating FAP in a sustained fashion in mice. We previously tested this compound in cynomolgus monkeys via bolus p.o. administration and achieved a transient decrease in FAP-related activity, consistent with the rapid clearance of this compound from circulation^29^. In this study, therefore, we tested the strategy of feeding cpd60-containing chow to mice and used the aP probe to track the resulting pharmacodynamics. The results indicated that cpd60 suppressed FAP activity in mice to undetectable levels. The ability of the aP probe to monitor changes in FAP activity without an influence of the residual PREP activity allowed us to demonstrate that cpd60 could completely suppress FAP at the dose we tested. We foresee that this feeding-based approach will promote pre-clinical investigation of the role of FAP to regulate endopeptidic substrates, such as α2AP and FGF21, as well as its role in diseases for which FAP has been implicated.

One of the most critical aspects of our new substrate probe described above is its ability to provide a strict FAP selectivity over highly related dipeptidyl peptidases such as DPPIV as well as more distally related endopeptidases such as PREP. To understand the mechanistic basis for the observed selectivity, we have compiled structural models of FAP, DPPIV and PREP with critical features of a peptide substrate included and experimentally substantiated these models. Our models highlight previously undescribed structural differences in substrate recognition by FAP and PREP, and the logic that enables recognition of peptides with a D-amino acid at P_2_ position by FAP but not by PREP.

In conclusion, we have developed a unique, accessible and specific homogenous assay for the endopeptidase activity of FAP. We propose that the assay presented here could guide preclinical investigations of FAP biology and the clinical development of FAP inhibitors or FAP-localized anti-tumor therapies in the future. As a complement to the *in vitro* assay, we are also investigating the potential utility of aP NIRF for *in vivo* imaging.

## Methods

### Ethical approval

All animal studies were conducted in accordance with the Guide for the Care and Use of Laboratory Animals, published by the National Institutes of Health (NIH) (NIH Publication 8523, revised 1985). The Institutional Animal Care and Use Committee (IACUC) at Genentech reviewed and approved all animal protocols. All the human samples were de-identified to investigators.

### Recombinant proteins

Human prolyl oligopeptidase (PREP, 4308-SE), DPPIV (1180-SE), DPP9 (5419-SE), and DPPII/DPP7 (3438-SE) proteins were purchased from R&D Systems. Human DPP8 (BML-SE527) protein was purchased from Enzo Life Sciences. N-terminal octa-His-tagged human and mouse FAP proteins containing the extracellular domain (residues L26-D760 and L26-D761, respectively) were purified from the conditioned media of transiently-transfected CHO cells, by immobilized metal affinity chromatography (IMAC), followed by size exclusion chromatography (SEC).

### Inhibitors

The FAP-specific inhibitor (S)-N-(2-cyano-4,4-difluoropyrrolidin-1-yl)-2-oxoethyl)-quinoline-4-carboxamide (cpd60) was synthesized according to the experimental procedure reported by Jansen *et al*.^43^. The PREP-specific inhibitor KYP-2047 was purchased from Sigma-Aldrich (St. Louis, MO).

### *In vivo* studies

Mice were maintained in a pathogen-free animal facility at 21 °C under standard 12 h light/12 h dark cycle with access to normal chow (Labdiet 5010) and water *ad libitum* unless otherwise indicated. Generation of *Fap* KO mice was described previously^30^. For *in vivo* pharmacological FAP inhibition, C57BL/6 background mice were fed *ad libitum* control chow (TD.00588, Envigo, Madison, WI), or the same diet containing 20 or 100 ppm of cpd60.

### Plasma and serum

Mouse plasma samples were prepared in K_3_-EDTA MiniCollect tubes (Greiner Bio-One, 450475) and frozen immediately at −80 °C before analysis. De-identified human serum samples were obtained from healthy donors through the Employee Donation Program at Genentech, Inc. The clinically diagnosed cirrhosis patient samples and healthy donor samples were purchased from Discovery Life Sciences (Los Osos, CA).

### FAP ELISA

Serum FAP concentration was measured using the human FAP DuoSet ELISA kit (R&D Systems, DY3715), according to the manufacturer’s protocol.

### Fluorescence resonance energy transfer (FRET)-quench assay

Peptides of the region flanking the C-terminal human FGF21 cleavage site (VGPSQG) or variants with D-amino acid at P2 were synthesized (Anaspec, Inc.), containing an amine-terminal donor (HyLite Fluor 488 or Cy5.5 for aP NIRF) and a dark quencher (QXL 520 or QSY21 for aP NIRF) conjugated to a supplementary C-terminal lysine. Additional peptides were generated as above, with the residue sequences shown in Fig. 1. ANP_FAP_^39^ and aP NIRF were synthesized by CPC Scientific (Sunnyvale, CA). Assays were conducted at 37 °C in 50 mM HEPES (pH 7.2), 150 mM NaCl, 1 mM EDTA, 0.1 mg/ml BSA. The excitation/emission wavelengths of cleaved peptide are 490/520 nm or 675/695 nm for ANP_FAP_ and aP NIRF. Fluorescence was monitored on a Tecan M1000 Pro plate reader in kinetic mode. Except where indicated otherwise, measurements of serum or plasma FAP activity were performed with the peptide substrate concentration at 6 µM and a 10-fold dilution of serum or plasma in assay buffer.

### Enzyme kinetics

Kinetics measurements were carried out using 5 nM human or mouse recombinant FAP, with a series of peptide substrate titrations. The initial rate of substrate hydrolysis was determined using the Magellan software on a Tecan M1000 Pro plate reader and kinetic parameters were modeled using nonlinear regression analysis with GraphPad Prism software. Standard error was calculated from three experimental replicates.

### Active site titration

The tight-binding FAP inhibitor, cpd60, was titrated into a GP peptide cleavage reaction with 100 nM of mouse or human recombinant FAP and 6 µM of GP. The IC_50_ of cpd60 for human and mouse FAP endopeptidase activity was previously determined to be <0.5 nM^30^. As described^51^, the active enzyme concentration was determined from the x-intercept.

### Statistics

Unpaired student’s t-test (two-tailed) was used for statistical analysis to compare treatment groups. A *p* value < 0.05 was considered statistically significant. All the values were presented as means ± SEM unless otherwise noted.

### Protein modeling

The Schrödinger Small-Molecule Drug Discovery Suite 2017-1, (Schrödinger, LLC, New York, NY, 2017) was used for modeling studies. Protein coordinate files were prepared using the default protein preparation workflow, including minimization of the coordinates using the OPLS3 force field^52,53^. Superposition of the IPI-peptide-bound coordinates of DPPIV (PDB Accession Number 1NU8) with those of apo FAP (PDB Accession Number 1Z68) allowed placement of the IPI peptide into the apo FAP structure. The FAP active site serine (S624) was replaced by alanine and the peptide-bound model of FAP was further refined by minimization. A model for PREP bound to an acetyl-VAPSQ-amide peptide was generated from peptide-bound PREP coordinates (PDB Accession Protein 1E8N); the peptide ligand was modified manually and the complex minimized. All structure figures were prepared using the PyMOL Molecular Graphics System, Version 1.8 (Schrödinger, LLC).

### Ligand docking calculations

The Schrödinger Induced Fit docking workflow was used to generate models of the acetyl-VaPSQ peptide bound to FAP^54^. The docking protocol employed a core restraint to localize the position of the proline in the active site (to within 0.5 Å of its location in the IPI peptide model) and a hydrogen bond restraint to the phenolic hydroxyl proton of Y541. FAP residues within 5 Å of the IPI ligand were allowed to move during the docking protocol, with the exception of several key residues involve in coordinating the substrate in the active site (R123, E203, Y541, A624 and H734). The docking grid was centered on the IPI peptide; default settings were used for all other aspects of the protocol. 20 final poses were generated.

### Data and materials availability

Datasets and materials generated during and/or analyzed during the current study are available from the corresponding author upon reasonable request.

## Electronic supplementary material

Supplementary Information
