# Supplementary material for: Selective Homogeneous Assay for Circulating Endopeptidase Fibroblast Activation Protein (FAP)
Source: Sci Rep. 2017 Oct 2;7:12524. doi: 10.1038/s41598-017-12900-8 (PMC5624913; doi:10.1038/s41598-017-12900-8)

Supplementary information.

Selective Homogeneous Assay for Circulating Endopeptidase Fibroblast Activation Protein (FAP)

Travis W. Bainbridge[1,+], Diana Ronai Dunshee[2,+], Noelyn M. Kljavin[3], Nicholas J. Skelton[4], Junichiro Sonoda[2,5,*] and James A. Ernst[1,6,*]

[1]Protein Chemistry, [2]Molecular Biology, [3]Molecular Oncology, [4]Discovery Chemistry, [5]Cancer Immunology, [6]Neuroscience, Genentech Inc., South San Francisco, CA, 94080, USA

Supplementary Figure 1. Selective inhibitors of FAP or PREP. (A) Cpd60, an FAP-selective inhibitor[43]. (B) KYP-2047, a PREP-specific inhibitor[44,45].

A

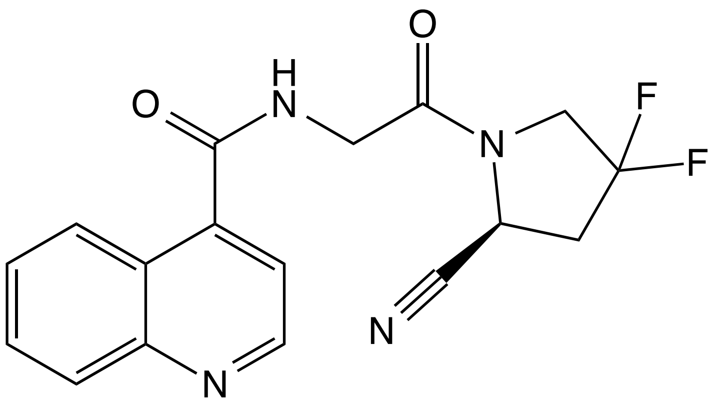

B

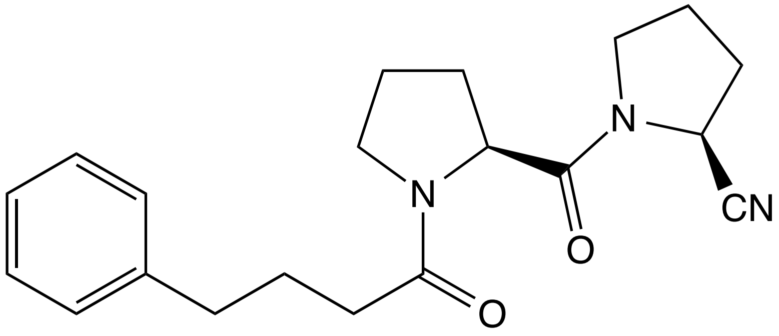

Supplement: Supplementary file 1 — Supplementary Information [file 41598_2017_12900_MOESM1_ESM.pdf]
